# Seven Strains of Enterovirus D68 Detected in the United States during the 2014 Severe Respiratory Disease Outbreak

**DOI:** 10.1128/genomeA.01201-14

**Published:** 2014-11-20

**Authors:** B. A. Brown, W. A. Nix, M. Sheth, M. Frace, M. S. Oberste

**Affiliations:** a Division of Viral Diseases, Centers for Disease Control and Prevention, Atlanta, Georgia, USA; b Division of Scientific Resources, Centers for Disease Control and Prevention, Atlanta, Georgia, USA

## Abstract

Clusters of severe respiratory disease in the United States were reported to the CDC beginning in August 2014. Enterovirus D68 (EV-D68) was identified from 83% (30/36) of initial severe cases. Investigations in August and September found severe EV-D68 cases to be widespread across the United States. We report seven EV-D68 genomes from the outbreak.

## GENOME ANNOUNCEMENT

Enteroviruses (EV) are small, nonenveloped viruses with a single-stranded, positive-sense RNA genome of 7.5 kilobases. The genus *Enterovirus* (family *Picornaviridae*) includes seven species that commonly infect humans: enterovirus A, B, C, and D and rhinovirus A, B, and C (http://www.picornaviridae.com/enterovirus/enterovirus.htm). EV-D68 was first identified in California in 1962 from cases of bronchiolitis and pneumonia (1). Since then, EV-D68 had been rarely reported in the United States until 2009, when several small clusters of severe respiratory disease were reported (2). The ongoing 2014 U.S. outbreak is the largest and most widespread EV-D68 outbreak investigated to date (3). Several EV-D68 strains have been identified cocirculating in the current outbreak. All cocirculating EV-D68 strains identified at CDC are represented in the seven EV-D68 genomes described.

One complete and six near-complete EV-D68 genome sequences were generated from RNA extracted directly from nasopharyngeal (NP) swab supernatants or from virus isolates obtained by inoculation of NP supernatants into human rhabdomyosarcoma cells (RD) (ATCC CCL-136). RNA from both sources was extracted using a QIAamp viral RNA kit (Qiagen). The RNA was used as the template in reverse transcription using the SMARTer Universal low-input RNA library prep kit (Clontech) with the modification that additional EV-specific 3′ and 5′ primers were supplemented. The first strand product was a template for amplification with the Advantage 2 polymerase mix (Clontech).

Amplification products were sheared to approximately 500 bp by sonication (Covaris). Sizes before and after shearing were evaluated on the Caliper LabChip GX analyzer (Perkin-Elmer). Sequencing libraries were prepared with the NEBNext Ultra DNA library prep kit for Illumina (New England Biolabs), employing unique sample bar coding. Sequencing was performed using the Illumina MiSeq Sequencing kit v. 2 (500 cycles). Five to twelve samples were pooled on a single flow-cell, generally yielding >2,718,000 reads/sample and average read lengths of 160 bp.

Contigs were first assembled with the *de novo* assembly feature in CLC Genomics software (v. 7.0.3) and yielded contig sequences averaging 6,900 nucleotides (nt). After “BLASTing” of contigs to the NCBI viral database, reads were then mapped to NCBI reference EF107098 or KF726085 and consensus sequences determined.

Consensus sequences mapped to reference yielded seven EV-D68 genomes, two from RD isolates, (lacking 2 and 24 nucleotides at the 5′ end) and five metagenomes from NP swabs, lacking from 0 to 449 nucleotides at the 5′ end. Complete coding sequences and 3′ untranslated regions (UTRs) were obtained for all seven genomes. Following the 5′ UTR, the single open reading frame (6,567 nt) encodes a polyprotein of 2,189 amino-acids for six of the genomes. A single EV-D68 genome encodes a polyprotein of 2,190 amino acids, with the open reading frame extended to 6,570 nt. The overall gene organization of the seven EV-D68s is identical to the defined taxonomic enterovirus pattern. The VP1 gene sequences of the seven EV-D68 strains are most closely related to EV-D68 viruses detected previously in recent years from the United States, Europe, and Asia.

### Nucleotide sequence accession numbers.

EV-D68 genomes were deposited in the GenBank database with the accession numbers KM851225 through KM851231.
